# Medium-chain fatty acids modify macrophage expression of metabolic and inflammatory genes in a PPAR β/δ-dependent manner

**DOI:** 10.1038/s41598-023-38700-x

**Published:** 2023-07-18

**Authors:** Paula V. Gaete, Luz D. Nieves-Barreto, Valentina Guatibonza-García, Mónica Losada-Barragán, Karina Vargas-Sánchez, Carlos O. Mendivil

**Affiliations:** 1 Department of Physiological Sciences, Universidad Nacional de Colombia, Bogotá, Colombia; 2 School of Medicine, Universidad de los Andes, Carrera 7 No 116-05, Of 413, Bogotá, Colombia; 3 Biología Celular y Funcional e Ingeniería de Moléculas, Departamento de Biología, Universidad Antonio Nariño, Bogotá, Colombia; 4 Section of Endocrinology, Department of Internal Medicine, Fundación Santa Fe de Bogotá, Bogotá, Colombia

**Keywords:** Cardiovascular diseases, Dyslipidaemias

## Abstract

There is great interest on medium chain fatty acids (MCFA) for cardiovascular health. We explored the effects of MCFA on the expression of lipid metabolism and inflammatory genes in macrophages, and the extent to which they were mediated by the nuclear receptor peroxisome proliferator-activated receptor beta/delta (PPAR β/δ). J774A.1 murine macrophages were exposed to octanoate or decanoate as MCFA, a long-chain fatty acid control (palmitate), or the PPAR β/δ agonist GW501516, with or without lipopolysaccharide (LPS) stimulation, and with or without an siRNA-induced knockdown of PPAR β/δ. MCFA increased the expression of *Plin2*, encoding a lipid-droplet associated protein with anti-inflammatory effects in macrophages, in a partially PPAR β/δ-dependent manner. Both MCFA stimulated expression of the cholesterol efflux pump ABCA1, more pronouncedly under LPS stimulation and in the absence of PPAR β/δ. Octanoate stimulated the expression of *Pltp*, encoding a phospholipid transfer protein that aids ABCA1 in cellular lipid efflux. Only palmitate increased expression of the proinflammatory genes *Il6, Tnf, Nos2* and *Mmp9.* Non-stimulated macrophages exposed to MCFA showed less internalization of fluorescently labeled lipoproteins. MCFA influenced the transcriptional responses of macrophages favoring cholesterol efflux and a less inflammatory response compared to palmitate. These effects were partially mediated by PPAR β/δ.

## Introduction

Cardiovascular diseases (CVD) are the first cause of mortality worldwide, being responsible for about 18 million deaths every year^1^. Atherosclerosis, the progressive narrowing of mid-size and large arteries by the continuous deposit of lipid and fibrous material, and by an inflammatory reaction in the vascular wall, is the pathophysiological mechanism underlying most of the clinical modalities of CVD^2^. Phagocytic cells, and macrophages in particular, are key players in the process that leads from the deposition lipoproteins in the arterial intima to the development of atherosclerotic plaques and their eventual rupture and complication^3^. Therefore, interventions aimed at modulating macrophage function are of high interest for the prevention of CVD. In particular, specific fatty acids may influence macrophage biology.

Fatty acids can be classified according to their length in short (up to 6 carbons), medium (7–12 carbons), or long chain (more than 12 carbons). Octanoic (C8) and decanoic (C10) acids, are saturated medium-chain fatty acids (MCFA) found in very low concentrations in the usual western diet^4^. Recent evidence has revealed that MCFA possess potentially beneficial anti-inflammatory^5^, anticonvulsant^6^, neuroprotective^7^, and anticarcinogenic^8^ effects. While any effects induced by MCFA likely involve multiple biological pathways, fatty acids are known to exert many of their actions trough binding to nuclear receptors^9^.

One group of such nuclear receptors is peroxisome proliferator-activated receptors (PPARs), a group of ligand-activated transcription factors whose activation results in a broad array of cellular responses. There are three types of PPARs, namely PPAR-alpha, PPAR-gamma and PPAR beta/delta (PPARβ/δ), all of which form heterodimers with the 9-cis retinoic acid receptor (RXR)^10^. Of note, despite similar ligand-binding domains, very small changes in the aminoacid composition of this domain among the three types of PPARs result in very different affinity for individual ligands^11^. While PPAR-alpha and PPAR-gamma have been more extensively studied due to the existence of agonists for clinical use, PPARβ/δ has been relatively less explored. Importantly, PPARβ/δ is the subtype most extensively expressed in macrophages^12^, and its activation in this cell type has been associated with changes in genes related to lipid metabolism, and with a generally anti-inflammatory response^13^.

Within this context, we wanted to explore the direct effect of MCFA on the expression of genes related to lipid metabolism and the inflammatory response in macrophages in vitro. Since MCFA may bind to and activate PPARs, and PPARβ/δ is the predominant subtype in macrophages, we also assessed to what extent the effects of MCFA on macrophage gene expression are mediated by PPAR β/δ.

## Methods

### Study design

This was an in vitro study of the response of macrophages to physiologically relevant concentrations of MCFA, and of the extent to which such responses were mediated by PPARβ/δ. Murine macrophages were exposed to octanoate or decanoate as MCFA, to palmitate (a long-chain fatty acid control), or to a specific PPAR-delta agonist (GW501516). GW501516 has a very high affinity (Km = 1 nM) for PPARβ/δ, with a > 1000-fold selectivity for PPARβ/δ over other PPARs^14^. The responses were measured under normal expression of *Ppard*, or after a short-interfering RNA(siRNA)-induced knockdown of *Ppard*. In order to assess the impact of macrophage activation on the transcriptional response to MCFA, the expression of multiple genes of interest was measured in the presence or absence of lipopolysaccharide (LPS). As a measure of the phagocytic activity of macrophages relevant to the pathogenesis of atherosclerosis, we also assessed the ability of macrophages to internalize fluorescently labelled apolipoprotein-B-containing lipoproteins (very-low density lipoproteins [VLDL] and low-density lipoproteins [LDL]). The overall design of the study is summarized in Table 1.

**Table 1:** Experimental design of the study.

	*Ppard* knockdown			
	Yes		No	
	LPS+	LPS−	LPS+	LPS−
Control	*	*	* †	* †
Octanoate	*	*	* †	* †
Decanoate	*	*	* †	* †
Palmitate	*	*	* †	* †
GW501516	*	*	* †	* †

*Analysis of the expression of genes associated with lipid and lipoprotein metabolism, and with the immune and inflammatory response.

*†Lipoprotein uptake assay. All assays were performed in triplicate.

### Cell culture

Mouse macrophages (J774A.1 line) were bought from the European Collection of Authenticated Cell Cultures (ECACC, CAT# 91051511), and thawed in Dulbecco’s Modified Eagle Medium (DMEM) supplemented with 20% fetal bovine serum (FBS), penicillin 100 UI/mL, streptomycin 100 µg/mL, amphotericin B 0.025 µg/mL and 2 mM l-glutamine. Cells were plated in T-25 flasks in a cabin with 5% CO_2_ at 37 °C, with medium changes every 24 h. After three days, cells were changed to maintenance medium (DMEM with 10% FBS, penicillin 100 UI/mL, streptomycin 100 µg/mL, amphotericin B 0.025 µg/mL and 2 mM l-glutamine). Later, cells were split in 6-well plates, at a density of 200,000 cells/well, with 3 mL of maintenance medium. One half of the cells underwent *Ppard* knockdown, and one half did not.

### Ppard knockdown

A small-interfering RNA (siRNA) system comprising 3 target-specific 19–25 nucleotides long siRNAs was used to knock down gene expression of *Ppard* (Santa Cruz Biotechnology, CAT SC-36306). Transfection of the siRNAs into macrophages was achieved using lipofectamine. The extent of *Ppard* knockdown was assessed 24–72 h after transfection using qPCR with *Gapdh* as normalizing gene. The sequence of the primers used were: Fw: 5′-GCACATCTACAACGCCTACC-3′ and Rv: 5′-GTGGATGACAAAGGGTGCG-3′ for *Ppard*, and Fw: 5′-TCAGGAGAGTGTTTCCTCGT-3′ and Rv: 5′-CCAATACGGCCAAATCCGTT-3′ for *Gapdh*.

### Exposure to treatments

Once the success of knockdown was verified, cells were changed to DMEM with 5% FBS and 2 mM l-Glutamine with or without 0.1 µg/mL LPS^15^ and incubated overnight. Next morning, cells were incubated in medium containing the study treatments according to the scheme presented in Table 1: No exposure (control), 250 µM octanoate, 250 µM decanoate, 250 µM palmitate (all concentrations comparable to those found in the plasma of human patients undergoing a medium-chain triglycerides [MCT]-rich diet)^16^, or 100 nM GW501516^17^. In addition, all culture media contained 5% FBS, penicillin 100 UI/mL, streptomycin 100 µg/mL, amphotericin B 0.025 µg/mL and 2 mM L-glutamine. Cells were exposed to these treatments for 72 h.

### RNA extraction and qPCR

After exposure to study treatments, cells were harvested, and their RNA was extracted with TRIzol reagent (1 mL per 5–10 million cells). The RNA was resuspended in nuclease-free water and its purity measured in a microvolume spectrophotometer. Reverse transcription was done using MultiScribe™ Reverse Transcriptase (Applied Biosystems CAT# 4368813), and random hexamer primers in the presence of RNAse inhibitor. Thermal cycler conditions were 25 °C for 10 min, 37 °C for 120 min and 85 °C for 5 min. For qPCR assays of gene expression, the template cDNA final concentration was 50 ng/µL, primers were 300 nM, and we used Applied Biosystem´s SYBR® Green PCR Master Mix (CAT# 4309155). The reference gene for normalization was *Gapdh*, relative gene expression was calculated using the 2^−∆∆Ct method. Primers for each gene of interest are shown in Table 2. Expression of each gene under each of the conditions was assayed in triplicate.

**Table 2:** Primers used for qPCR expression analysis of the genes of interest.

Gene	Forward primer	Reverse primer	Amplicon size
*Ppard*	GCACATCTACAACGCCTACC	GTGGATGACAAAGGGTGCG	103
*Scarb1*	TTTGGAGTGGTAGTAAAAAGGGC	TGACATCAGGGACTCAGAGTAG	71
*Plin2*	GTCCCTCAGCTCTCCTGTTA	CTCATCACCACGCTCTGTTG	91
*Npc1*	GTCACCTACGTGGCATTTCT	CACAAAGTACCGCCTTCTGT	78
*Pltp*	CAATATCTCGGACGTGAGGG	TTCAGCAGCAGATCTTGGTC	84
*Cpt1a*	GACTCCGCTCGCTCATTC	TCTGTTTGAGGGCTTCATGG	145
*Abca1*	GTGGAATCGTCCCTCAGTTC	CTGAGAAACACTGTCCTCCTTT	78
*Abcg1*	AAGGTTGCCATAGCTTCTCG	CAGATGTGTCAGGACCGAGT	101
*Il6*	GACAAAGCCAGAGTCCTTCAG	TGTGACTCCAGCTTATCTCTTG	76
*Il10*	CTGTCATCGATTTCTCCCCTG	CACCTTGGTCTTGGAGCTTATTA	86
*Tnf*	CCCTCACACTCACAAACCAC	TTTGAGATCCATGCCGTTGG	92
*Ifng*	TCAAGTGGCATAGATGTGGA	TTTCATGTCACCATCCTTTTGC	78
*Nos2*	AGCGCTCTAGTGAAGCAAAG	AGGGATTCTGGAACATTCTGTG	107
*Ptgs2*	TCCAACCTCTCCTACTACACC	AGGAAGCTCCTTATTTCCCTTC	90
*Ccl2*	TTCACCAGCAAGATGATCCC	TGAGCTTGGTGACAAAAACTAC	100
*Mmp9*	AGCCGACTTTTGTGGTCTTC	GCGGTACAAGTATGCCTCTG	83
*Gapdh*	TCAGGAGAGTGTTTCCTCGT	CCAATACGGCCAAATCCGTT	70

### Lipoprotein uptake assay

In order to assess how gene expression changes translated into phagocytic activity of relevance to cardiovascular disease, we performed a lipoprotein uptake assay in which the internalization of fluorescently labelled very-low-density lipoproteins (VLDL) and low-density lipoproteins (LDL) by macrophages under the different conditions was measured. For the separation of a fraction containing both VLDL and LDL, blood from a fasting healthy volunteer was drawn in EDTA tubes, and plasma was promptly separated and kept chilled in ice. A density gradient was prepared in 1 mL ultracentrifuge tubes by adding 200 µL of a 48% sucrose solution, then 285 µL of plasma with 4 M NaCL, and lastly 514 µL of a 0.67 M NaCl, 0.5% EDTA solution^18^. Samples were then centrifuged in a S140-AT rotor inside a Sorvall MTX-150 ultracentrifuge at 86,260 rpm, 10 °C for 105 min. The first 20 fractions out of 44, corresponding to the top 454 µL in the surface of the tube, were carefully collected to avoid perturbing the gradient. The appropriate separation of VLDL and LDL was confirmed by separating and visualizing them in a 2% agarose-TBE gel, with Sudan staining. The lipoprotein solution was then incubated overnight with 2 µM 4,4-difluoro-4-bora-3a,4a-diaza-s-indacene (BODIPY) at 37 °C, in the dark. Next morning the unbound fluorophore was discarded by washing the labelled lipoproteins three times with PBS in a 10 KDa concentrator tube. After this, the lipoprotein extract was reconstituted to its initial volume with PBS.

J774A.1 macrophages were plated in 96-well culture plates at a density of 10,000 cells/well, in maintenance medium, for 24 h. Culture medium was then replaced by fresh medium with or without LPS, and cells were incubated another 16 h. Then, cells were incubated for 24 h at 37 °C, 5% CO_2_ in a mixture of 50% maintenance medium with each of the study exposures, and 50% lipoprotein fraction containing VLDL and LDL. Cells were exposed to a final apoB concentration of 0.385 g/L, corresponding to an apoB (and hence VLDL + LDL) molar concentration of 0.7 mM. Afterwards, the medium from each well was carefully aspirated and placed in a clean well. The fluorescence (excitation 495 nm, emission 507 nm) was measured in the well with the cells and in the well with the medium, and the percent lipoprotein uptake was calculated as: fluorescence in cells/(fluorescence in cells + fluorescence in medium).

### Statistical analysis

Group means of continuous variables were compared using a three-way linear model (ANOVA), with treatment (control, octanoate, decanoate, palmitate or GW501516), *Ppard* knockdown (Y/N), and LPS activation (Y/N) as fixed effects. Simple contrasts were used to compare means between levels of each factor. The hypothesis that the effect of treatments on gene expression differed depending on the knockdown of *Ppard* was tested with the p-value associated with the siRNA*treatment interaction term in the ANOVA. For all analyses presented in figures, the reference group with a gene expression equivalent to one was that of macrophages not exposed to any treatment, not stimulated with LPS, and without the knockdown for *Ppard*. The significance level for all statistical comparisons was 0.05 (two-tailed). Analyses were performed in SPSS Statistics v24.

### Ethical aspects

The project was approved by the IRB of Universidad Nacional de Colombia, according to minute 004-034 of 2022. All methods were carried out in accordance with relevant guidelines and regulations. Informed consent was obtained from all subjects and/or their legal guardian(s).

### Ethics approval and consent to participate

This study was approved by the Ethics Committee of the School of Medicine, National University of Colombia, according to minute 004-034 of March 10, 2022.

## Results

**Figure 1:**
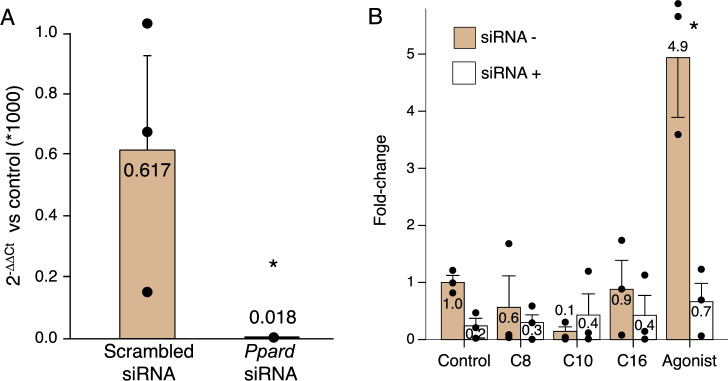
Expression of *Ppard* by murine macrophages after siRNA, and after exposure to the study treatments. (**A**) There was an average 97% reduction in the expression of Ppard with the use of the siRNA. (**B**) *P* = 0.043 for the overall difference in *Ppard* expression with *versus* without the siRNA. *Significant difference in effect *versus* the control group.

With the use of the siRNA, we obtained an average 97% *knockdown* in the expression of *Ppard* in murine macrophages (Fig. 1, Panel A). Exposure to any of the studied fatty acids caused non-significant reductions in the expression of *Ppard*, while exposure to GW501516 increased it (Fig. 1, Panel B).

### Effects on genes related to lipid uptake, storage and efflux

**Figure 2:**
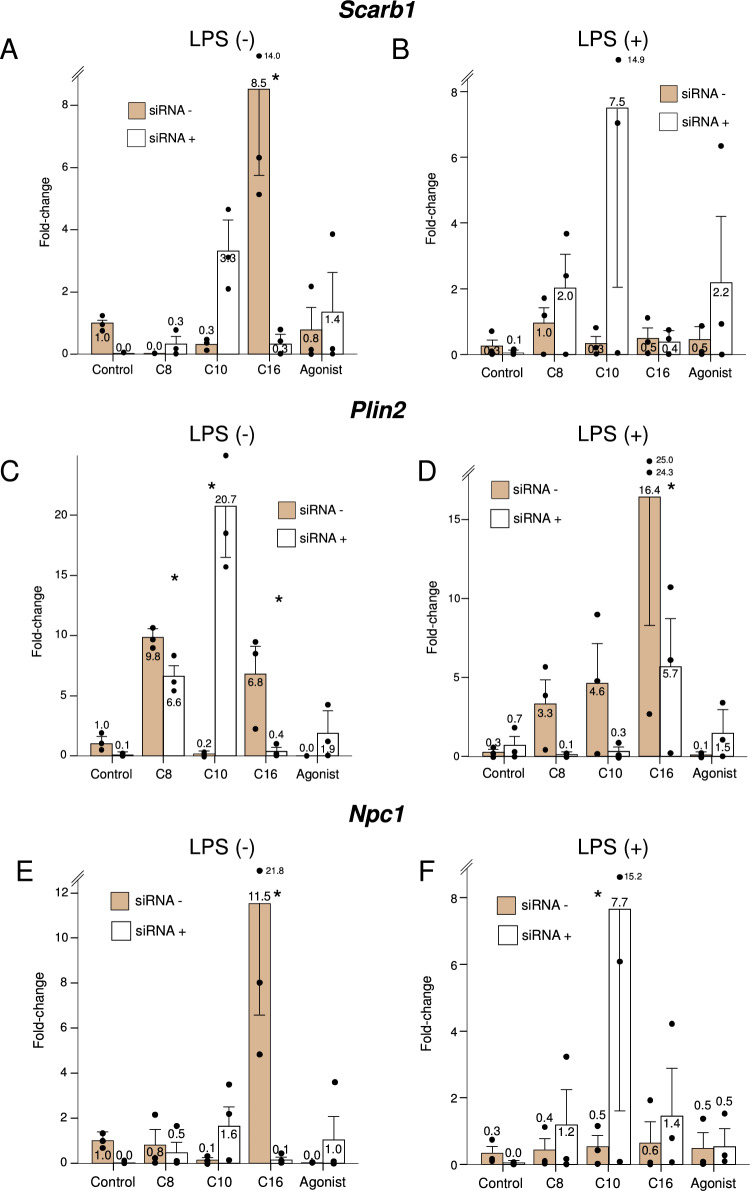
Expression of genes related to cellular uptake, storage, and efflux of lipids, under the study treatments, in the presence (dark bars) or knockdown (light bars) of *Ppard,* in resting or LPS-stimulated murine macrophages. C8: Octanoate, C10: Decanoate, C16: Palmitate, Agonist: GW501516. Bars represent fold-change relative to the control group without *Ppard* knockdown, and without LPS stimulation. Error bars represent the standard error of the mean. *Significant difference in treatment effect *versus* the control group. *P*-value for the siRNA*treatment interaction term: 0.009 for *Scarb1*, 0.001 for *Plin2* and 0.008 for *Npc1*.

*Scarb1* encodes the scavenger receptor 1, a key protein in the flux of cholesterol to and from lipoproteins. Neither octanoate nor decanoate induced statistically significant changes in the expression of *Scarb1*, in either resting or LPS-stimulated macrophages. However, there was a trend towards increased *Scarb1* expression with decanoate treatment in macrophages with the *Ppard* knockdown, suggesting the loss of a repressive effect when *Ppard* is absent. Meanwhile, palmitate stimulated *Scarb1* expression only in the presence of *Ppard*, the effect being larger in non-stimulated macrophages (Fig. 2, panels A and B). *Plin2* encodes perilipin 2, a protein found in the periphery of intracellular lipid droplets and involved in lipid storage. Both octanoate and decanoate significantly increased the expression of *Plin2* in non-stimulated macrophages (*P* < 0.001 in both cases), albeit for decanoate the effect was present only with *Ppard* knockdown (*P* = 0.001 for the siRNA/treatment interaction). The effect was at least partially dependent on the presence of *Ppard* (Fig. 2, panels C and D). Meanwhile, palmitate increased the expression of *Plin2* in LPS-stimulated macrophages (*P* = 0.002), an effect that was greatly attenuated by *Ppard* knockdown. *Npc1* encodes the NPC cholesterol transporter (its human ortholog is implicated in Niemann-Pick disease type C1), a membrane cholesterol transporter. In the absence of LPS stimulation, only palmitate increased expression of *Npc1*. Under stimulation, exposure to decanoate in the absence of *Ppard* also resulted in marked *Npc1* upregulation (Fig. 2, panels E and F).

**Figure 3:**
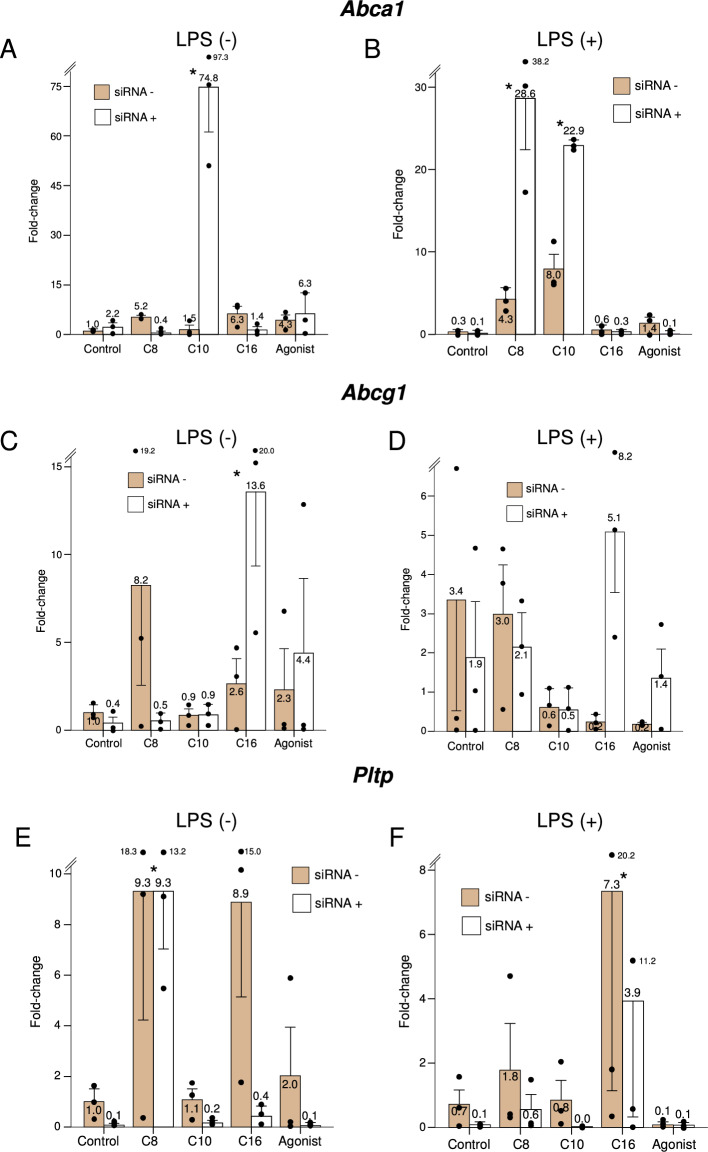
Expression of genes related to lipoprotein-mediated cholesterol transport under the study treatments, in the presence (dark bars) or knockdown (light bars) of *Ppard,* in resting or LPS-stimulated murine macrophages. C8: Octanoate, C10: Decanoate, C16: Palmitate, Agonist: GW501516. Bars represent fold-change relative to the control group without *Ppard* knockdown, and without LPS stimulation. Error bars represent the standard error of the mean. *Significant difference in treatment effect *versus* the control group. P-value for the siRNA*treatment interaction term: < 0.001 for *Abca1*, 0.10 for *Abcg1* and 0.65 for *Pltp*.

*Abca1* encodes the ATP-binding cassette transporter ABCA1, which works as a cholesterol efflux pump that aids in the removal of cholesterol from macrophages. In resting macrophages, decanoate strongly induced overexpression of *Abca1*, albeit only in the absence of *Ppard*. Under LPS stimulation, both octanoate and decanoate promoted Abca1 expression, mostly when *Ppard* was knocked down (Fig. 3, panels A and B). *Abcg1* encodes the ATP-binding cassette transporter ABCG1, related to ABCA1 but specifically involved in the sorting of intracellular sterols to different compartments^19^. *Abcg1* was modulated significantly only by palmitate, which increased its expression in resting macrophages when *Ppard* was not present (Fig. 3, panel C). We observed an increased expression of *Abcg1* with octanoate in the unstimulated state in the presence of *Ppard*, but it did not reach statistical significance. *Pltp* encodes the phospholipid transfer protein PLTP, which mediates the transfer of phospholipids between lipoproteins, and may have anti-inflammatory effects in macrophages^20^. Octanoate stimulated the expression of *Pltp* in resting macrophages, independently of *Ppard,* while palmitate stimulated it under LPS stimulation, and to a lower extent (Fig. 3, Panels E and F).

**Figure 4:**
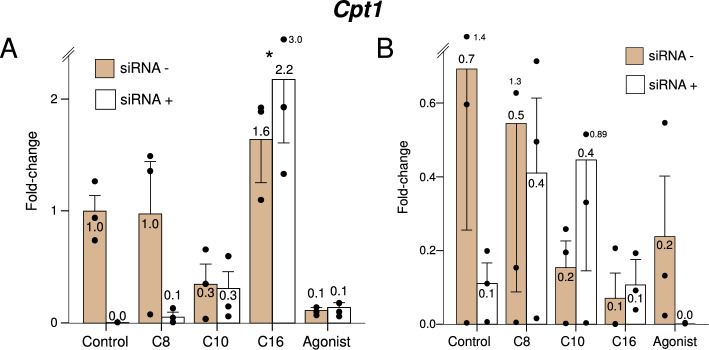
Expression of *Cpt1* under the study treatments, in the presence (dark bars) or knockdown (light bars) of *Ppard,* in resting or LPS-stimulated murine macrophages. C8: Octanoate, C10: Decanoate, C16: Palmitate, Agonist: GW501516. Bars represent fold-change relative to the control group without *Ppard* knockdown, and without LPS stimulation. *Significant difference in treatment effect *versus* the control group. *P*-value for the siRNA*treatment interaction term: 0.15.

*Cpt1* encodes carnitine-palmitoyl transferase 1 (CPT-1), a key enzyme in the transport of long-chain fatty acids into the mitochondria for their beta-oxidation. Concordant with the known function of CPT-1, expression of this gene was induced only by palmitate, and only in the non-stimulated state (Fig. 4, panel A).

### Effects on genes related to immune function and the inflammatory response

**Figure 5:**
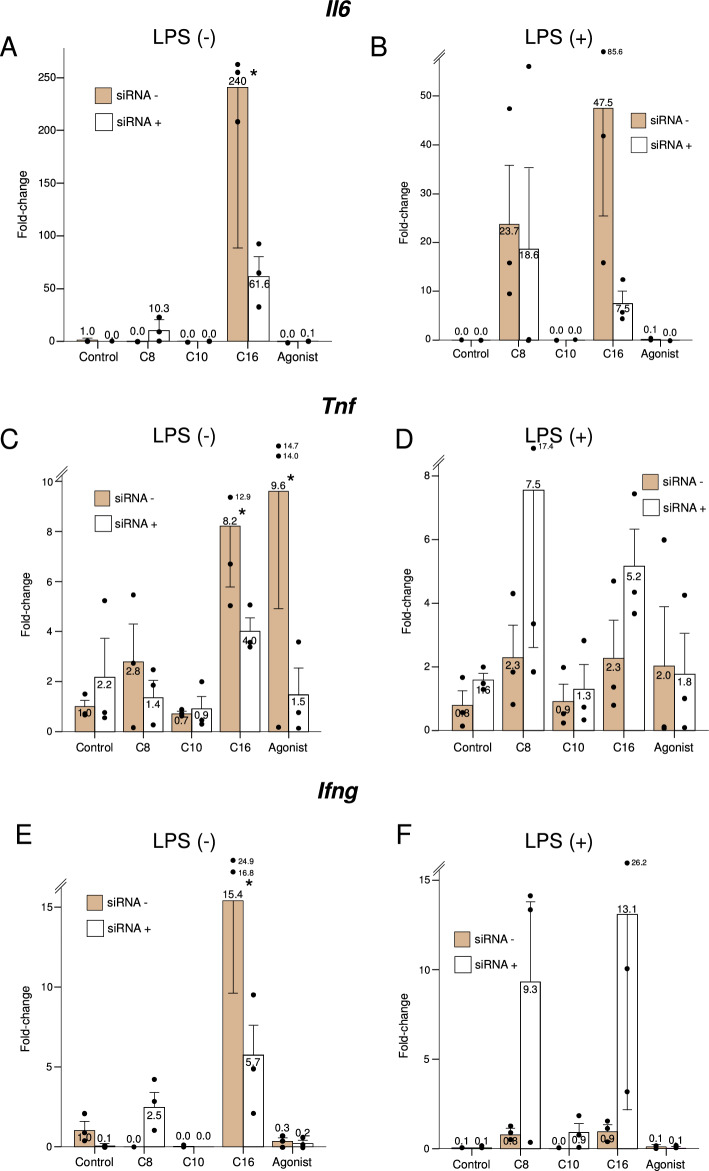
Expression of the genes for proinflammatory cytokines under the study treatments, in the presence (dark bars) or knockdown (light bars) of *Ppard,* in resting or LPS-stimulated murine macrophages. C8: Octanoate, C10: Decanoate, C16: Palmitate, Agonist: GW501516. Bars represent fold-change relative to the control group without *Ppard* knockdown, and without LPS stimulation. Error bars represent the standard error of the mean. *Significant difference in treatment effect *versus* the control group. P-value for the siRNA*treatment interaction term: < 0.001 for *Il6*, 0.23 for *Tnf* and 0.89 for *Ifng*.

In general, the effect of study treatments on inflammatory genes was difficult to evidence in LPS-stimulated macrophages, due to the marked overall increase in cytokine expression under this condition. Palmitate substantially induced expression of *Il6* (encoding interleukin 6), in a manner partially dependent of *Ppard* (Fig. 5, Panel A)*.* Octanoate seemed to also increase *Il6* expression to a lesser extent, but this did not reach statistical significance. Palmitate also induced the expression of *Tnf* (encoding tumor necrosis factor-alpha) (Fig. 5, Panel C), and tended to increase expression of *Ifng* (encoding interferon-gamma) (Fig. 5, Panels E and F). Interestingly, GW501516 increased the expression of *Tnf* in non-stimulated macrophages, in a *Ppard*-dependent manner.

None of the study treatments affected the expression of *Ccl2*, which encodes Monocyte Chemoattractant Protein 1 (MCP-1), in either resting or stimulated macrophages. Similarly, no significant differences were found with any treatment in the expression of *Il10*, which encodes interleukin 10, a strongly anti-inflammatory cytokine.

**Figure 6:**
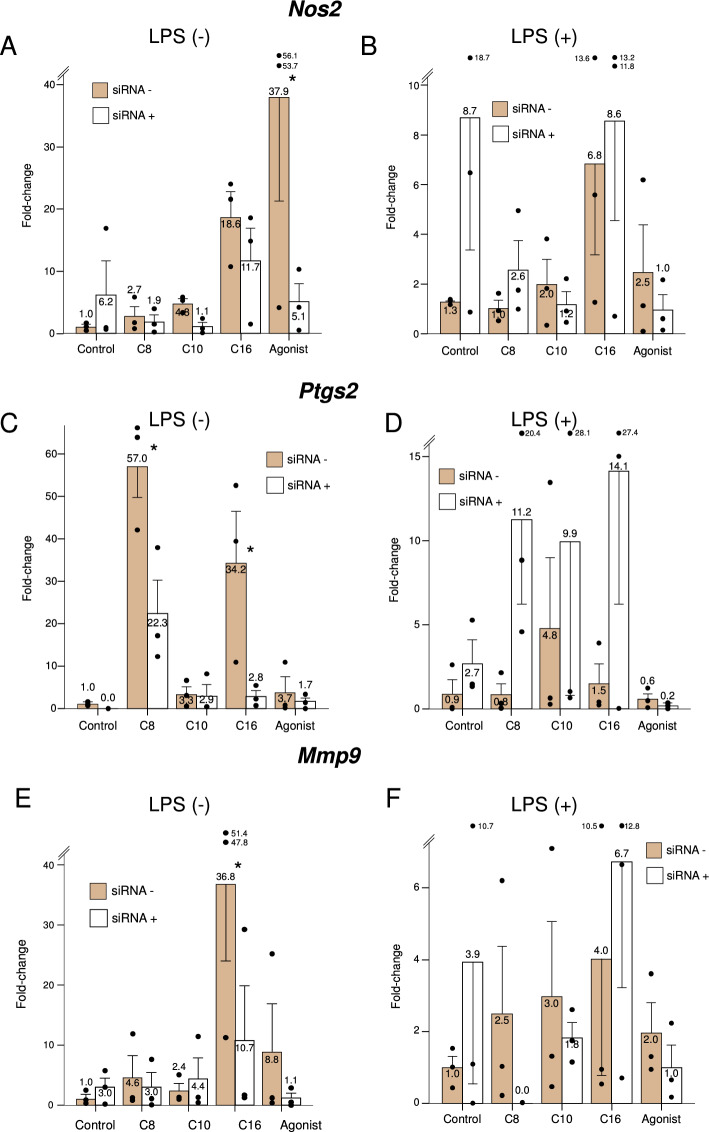
Expression of genes involved in macrophage activation under the study treatments, in the presence (dark bars) or knockdown (light bars) of *Ppard,* in resting or LPS-stimulated murine macrophages. C8: Octanoate, C10: Decanoate, C16: Palmitate, Agonist: GW501516. Bars represent fold-change relative to the control group without *Ppard* knockdown, and without LPS stimulation. Error bars represent the standard error of the mean. *Significant difference in treatment effect *versus* the control group. *P*-value for the siRNA*treatment interaction term: 0.02 for *Nos2*, 0.33 for *Ptgs2* and 0.30 for *Mmp9*.

*Nos2* encodes inducible nitric oxide synthase, an enzyme required for the production of free oxygen radicals by activated macrophages. Only palmitate induced *Nos2*, and only in the presence of *Ppard* (Fig. 6, Panel A). *Ptgs2* encodes prostaglandin-endoperoxide synthase 2, also known as cyclooxygenase type 2 (COX-2), an enzyme that catalyzes a common first step in the biosynthesis of multiple prostaglandins. Both octanoate and palmitate induced *Ptgs2* expression, in both cases in a *Ppard*-dependent fashion, and only in non-stimulated cells (Fig. 6, Panel C). *Mmp9* encodes matrix metallopeptidase 9, an enzyme in charge of degrading extracellular matrix components enabling macrophage tissular mobility. Only palmitate induced *Mmp9*, and only in resting cells, in a *Ppard*-dependent fashion (Fig. 6, Panel E).

In order to facilitate comparisons, the effect of octanoate, decanoate and palmitate on the expression of the analyzed genes is summarized in Table 3.

**Table 3:** Summary of the effects of octanoate, decanoate and palmitate on the expression of metabolic and inflammatory genes.

	LPS (−)			LPS (+)		
	Octanoate	Decanoate	Palmitate	Octanoate	Decanoate	Palmitate
Metabolic genes						
*Ppard*	=	=	=	=	=	=
*Scarb1*	=	=	↑*	=	=	=
*Plin2*	↑*	↑↑*	↑*	=	=	↑*
*Npc1*	=	=	↑*	=	↑*	=
*Abca1*	=	↑↑↑*	=	↑↑*	↑↑*	=
*Abcg1*	=	=	↑	=	=	=
*Pltp*	↑	=	=	=	=	↑
*Cpt1*	=	=	↑	=	=	=
Inflammatory genes						
*Il6*	=	=	↑↑↑↑*	=	=	=
*Tnf*	=	=	↑	=	=	=
*Ifng*	=	=	↑	=	=	=
*Mcp1*	=	=	=	=	=	=
*Il10*	=	=	=	=	=	=
*Nos2*	=	=	=	=	=	=
*Ptgs2*	↑↑	=	↑	=	=	=
*Mmp9*	=	=	↑↑	=	=	=

*The effect differed significantly in the presence vs absence of *Ppard* knockdown.

### Lipoprotein uptake assay

**Figure 7:**
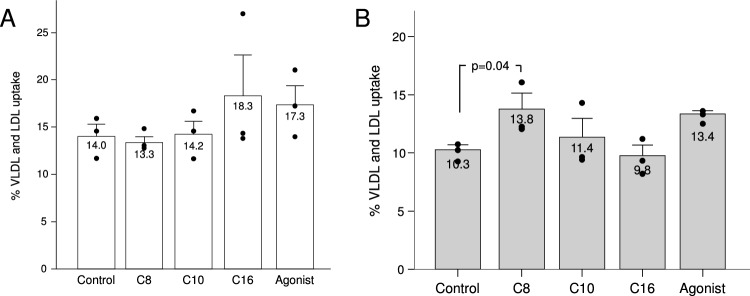
Uptake of labelled VLDL and LDL by murine macrophages under the study treatments, in resting (**A**) or LPS-stimulated (**B**) murine macrophages. C8: Octanoate, C10: Decanoate, C16: Palmitate, Agonist: GW501516. Bars represent the proportion of total labeled VLDL + LDL that was taken up by the cells. Error bars represent the standard error of the mean.

In resting conditions, the uptake of VLDL + LDL in the octanoate and decanoate groups was similar to the control, and numerically lower than that of palmitate or the agonist, but the difference was not statistically significant. Meanwhile, under LPS stimulation octanoate induced a significantly larger lipoprotein uptake (Fig. 7).

## Discussion

In this study we found that MCFA can substantially influence the expression of genes involved in lipid metabolism and the inflammatory response in macrophages. A considerable part of the observed effects appeared to be mediated by the nuclear receptor PPARβ/δ. Given the essential involvement of macrophages and their conversion to foam cells in the development and progression of atherosclerosis, these results provide relevant information about the potential of MCFA for CVD prevention.

We found that despite being generically grouped together under the umbrella term “medium-chain fatty acids”, the cellular response to octanoate and decanoate was different, especially concerning the observed modulatory effects on lipid metabolism genes. Previous studies have documented that the transcriptional response after exposure to individual fatty acids is highly dependent on both the fatty acid length and the type of cell being exposed^21^. The use of palmitate as a control allowed us to reveal effects specific to MCFA, and not common to all saturated fatty acids.

Interestingly, decanoate induced expression of the gene for the macrophage scavenger receptor SR-B1 (*Scarb1)*,—which in macrophages plays a fundamental role in the process of cholesterol flux to and from high-density lipoproteins (HDL)^22^—only when *Ppard* was knocked down. Thus, it is possible that under normal conditions, binding of decanoate to PPAR β/δ inhibits expression of SR-B1, or that decanoate binds to a different receptor whose effect on *Scarb1* is stimulatory, with less affinity than to PPAR β/δ. Since SR-B1 facilitates the docking of HDL to macrophages and the uptake by HDL of free cholesterol for later esterification, this effect might favor the process of reverse cholesterol transport.

The role of perilipin 2 (encoded by *Plin2*), a lipid droplet-associated protein, in mononuclear cells, is not completely understood; but prior studies have reported that knockdown of perilipin 2 in THP-1 macrophages is associated with increased lipid droplet size and reduced inflammatory response^23^. Even though both octanoate and decanoate (when *Ppard* was knocked down) significantly increased the expression of *Plin2* in non-stimulated macrophages, they both did so to a much lesser extent than palmitate at the same concentration, suggesting that the substitution of MCFA for longer chain fatty acids may have a net anti-inflammatory impact. Studies in *Npc1* knockout mice had described an antiatherogenic effect of the Niemann-Pick C1 transporter through its ability to induce the formation 27-hydroxysterols, which may serve as ligands for liver-X receptors (LXR) and promote cholesterol efflux from macrophages^24^. We found that decanoate stimulated *Npc1*, but only when *Ppard* was absent. Thus, our results do not suggest a positive impact of MCFA on this component of the intracellular cholesterol trafficking machinery.

Both of the studied MCFA stimulated the expression of the gene for the ATP-binding cassette transporter A1 (ABCA1), most strongly when macrophages were stimulated by LPS, and when *Ppard* was not present. ABCA1 acts a mediator in the efflux of macrophage cholesterol and phospholipids to apolipoprotein A-I (apoA-I)^25^, and its overexpression prevents the development of atherosclerosis in LDL receptor-knockout mice^26^. ABCA1 is considered essential for the first step of the reverse cholesterol transport mechanism. Furthermore, prior studies in murine models have found that the anti-inflammatory effects of MCTs are accompanied by increased expression of ABCA1^27^, and absent or attenuated in ABCA1 deficiency^28^. In a very relevant experiment exploring the influence of medium chain fats on reverse cholesterol transport, Zhang et al. randomized apoE-deficient mice to receive a diet with 2% MCT or 2% long-chain triglycerides (LCT) as controls. The MCT group had an improved serum lipid profile and experienced a smaller burden of atherosclerotic plaques. Liver gene expression profiles showed higher expression of ABCA1, and of other genes implicated in reverse cholesterol transport (*Abcg*5, *Cyp7a1*) in the MCT group^29^. A follow-up study demonstrated that these anti-atherosclerosis effects of octanoate were attributable to modulation of the TLR4/NFkB pathway^30^.

We also found that octanoate stimulated the expression of the gene for the phospholipid transfer protein (PLTP). There is evidence that PLTP produced by macrophages contributes to ABCA1-dependent cholesterol efflux, as cholesterol-laden macrophages from PLTP-knockout mice have significantly impaired release of cholesterol to apoA-I, an effect that is rescued by the addition of cyclic AMP, an activator of ABCA1^31^. By stimulating the expression of the genes for both ABCA1 and PLTP, octanoate has the potential to favor cholesterol extrusion from cells. So, our results strengthen the case for a positive impact of MCFA on the efflux of cholesterol from macrophages.

While in general we did not find a robust effect of MCFA on the expression of genes related to the inflammatory and immune response, we did observe a systematically pro-inflammatory effect of palmitate. Palmitate increased the expression of the genes for IL-6, TNF-alpha, IFN-gamma, NOS2 and MMP-9, all of them implicated in the inflammatory response through different pathways. As discussed above, this pattern suggests that equimolar substitution of MCFA for palmitate may result in a net anti-inflammatory effect. This idea has been supported by studies in which mice fed diets with different fat composition have been compared in their ability to mount an IL-6 and TNF-alpha response to an LPS challenge^32^. MCT-rich diets resulted in the lowest response, and MCT were able to partially counter the inflammatory response induced by other fat types. The only gene of this group that was directly influenced by MCFA was *Ptgs2*, we found that octanoate and decanoate induced it only in the presence of *Ppard*. This is not necessarily a pro-inflammatory action, as COX-2 catalyzes a common step in the synthesis of both pro- and anti-inflammatory eicosanoids^33^. The mediation of PPAR β/δ in this effect of MCFA is in line with prior experiments showing that PPAR β/δ is important for the polarization of macrophages towards the anti-inflammatory M2 phenotype, characterized by reduced activity of the NF-kB pathway^34^.

In our lipoprotein uptake assays, there was a trend towards lower VLDL + LDL uptake in unstimulated cells exposed to MCFA which did not reach statistical significance. The finding of comparatively higher lipoprotein uptake in LPS-activated macrophages exposed to octanoate can be due to actually enhanced phagocytic activity under these circumstances but must be analyzed in the context of the simultaneous increase in cholesterol efflux genes.

It is important to bear in mind that, while we centered our analysis on the mediation by PPAR β/δ of the impact of MCFA on macrophages, many different fatty acid receptors can be expressed by cells of the immune system, among them FFAR1, FFAR2, FFAR3, FFAR4, GPR84, GPR109A, GPR170, GPR31, GPR132 and GPR119; and the activation of these receptors is able to modulate the macrophage inflammatory response^35^. Furthermore, fatty acids may also exert transcriptional effects by receptor-independent mechanisms^36^. In any event, prior analyses based on machine learning algorithms to identify potential target genes of PPARs predict *Scarb1, Plin2, Pltp, Abca1, Cpt1, Il6* and *Nos2* to be PPAR β/δ—responsive genes^37^. A relevant limitation of our study is that we did not directly measure the protein concentrations of the PPAR β/δ transcription factor. Future directions for research that would expand on our results include studying of the impact of MCTs on functional activity assessed with a cholesterol efflux system based on lipid-free apolipoproteins or mature HDL; as well as confirming out findings in a human monocytic cell line. Also, our results suggest that it would be important to study the extent to which MCTs may enhance SR-B1-dependent cholesterol uptake by hepatocytes.

In summary, we found that MCFA are able to influence the transcriptional responses of macrophages, in ways that may favor cholesterol efflux and, by comparison with longer chain fatty acids, an anti-inflammatory phenotype. These results add to the ongoing research effort towards understanding the potential clinical applications of MCFA and MCT-rich ketogenic diets.

## Data Availability

The datasets generated during and/or analyzed during the current study are available from the corresponding author on reasonable request.
